# The use of minimum selectable concentrations (MSCs) for determining the selection of antimicrobial resistant bacteria

**DOI:** 10.1007/s10646-017-1762-y

**Published:** 2017-02-02

**Authors:** Sadia Khan, Tara K. Beattie, Charles W. Knapp

**Affiliations:** 1 Department of Civil and Environmental Engineering, University of Strathclyde, James Weir Building, 75 Montrose Street, Glasgow, Scotland G1 1XJ UK; 2 Department of Environmental Engineering, NED University of Engineering and Technology, University Road, Karachi, 75270 Pakistan

**Keywords:** Minimal selective concentration (MSC), Minimum inhibitory concentration (MIC), Disinfectant, Drinking water

## Abstract

The use of antimicrobial compounds is indispensable in many industries, especially drinking water production, to eradicate microorganisms. However, bacterial growth is not unusual in the presence of disinfectant concentrations that would be typically lethal, as bacterial populations can develop resistance. The common metric of population resistance has been based on the Minimum Inhibitory Concentration (MIC), which is based on bacteria lethality. However, sub-lethal concentrations may also select for resistant bacteria due to the differences in bacterial growth rates. This study determined the Minimal Selective Concentrations (MSCs) of bacterial populations exposed to free chlorine and monochloramine, representing a metric that possibly better reflects the selective pressures occurring at lower disinfectant levels than MIC. Pairs of phylogenetically similar bacteria were challenged to a range of concentrations of disinfectants. The MSCs of free chlorine and monochloramine were found to range between 0.021 and 0.39 mg L^−1^, which were concentrations 1/250 to 1/5 than the MICs of susceptible bacteria (MIC_*susc*_). This study indicates that sub-lethal concentrations of disinfectants could result in the selection of resistant bacterial populations, and MSCs would be a more sensitive indicator of selective pressure, especially in environmental systems.

## Introduction

Overuse and misuse of antimicrobials during the last century have created issues related to the emergence and enrichment of resistant bacteria (Carlet et al. 2012), especially antimicrobial resistant pathogens that could contaminate water supply systems and survive their disinfection (Khan et al. 2016b; Xu et al. 2016). Almost every antibiotic has bacteria that have shown resistance to it (Kummerer 2009b), and these bacteria have been found in water, sanitation and agricultural industries (Kummerer 2009a; Li et al. 2016) and could be attributed to selective pressures exerted by environmental concentrations of antimicrobials (Tello et al. 2012; Sandegren 2014). There are concerns that these selective pressure will increase the evolution and spread of antibiotic resistant pathogens (Baquero et al. 1998; Bengtsson-Palme and Larsson 2016), and that the driving forces behind the development and selection of resistance are not fully understood due to the complexity of the interactions between bacteria, antimicrobials and environment.

Minimum Inhibitory Concentration (MIC) has been used widely to understand the susceptibility and resistance of bacteria to antimicrobials; this was derived in the clinical setting to represent population lethality. Basically, resistant populations become selected at environmental concentrations higher than the MIC of susceptible bacteria (MIC_*susc*_), while sub-MIC levels allow the continued growth of both susceptible and resistant genotypes (Andersson and Hughes 2014, Bengtsson-Palme et al. 2014a). Traditionally, it has been presumed that resistant bacteria have a competitive advantage at concentrations greater than the MIC (Sandegren 2014).

**Fig. 1:**
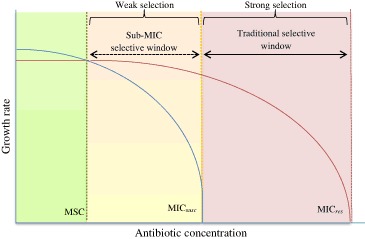
Schematic representation of growth rates as a function of antibiotic concentrations. *MICsusc* (*blue line*) minimum inhibitory concentration for susceptible strain, *MICres* (*red line*) minimum inhibitory concentration for resistant strain, *MSC* minimum selective concentration. Adapted from Gullberg et al. (2011) and Sandegren (2014) (color figure online)

However, concentrations below the MIC could favour highly resistant bacteria (Li et al. 2016). As such, microbiologists have defined minimum selective concentrations (MSC), which represents the lowest concentration of antimicrobials that gives the resistant strains a competitive advantage based on growth rates (Fig. 1) (Andersson and Hughes 2014). This better reflects enrichment possibilities of resistant bacteria in environments where low levels of antimicrobial are present, for example in soils and drinking water sources (Baquero et al. 1998, 2008; Fram and Belitz 2011; Jiang et al. 2013; Khan et al. 2013).

The MSC represents the point at which the benefit in growth exceeds the cost (fitness cost) of carrying the resistance trait vs. a non-resistant strain (Gullberg et al. 2011), and as such, there is a competitive advantage for having the resistance trait at concentrations greater than MSC (Sandegren 2014). The difference in fitness between susceptible and resistant organisms at sub-MIC values could result in toxicological endpoints (MSC) much lower than MIC against some antimicrobials (Liu et al. 2011).

The resistant populations selected at sub-MIC concentrations could pose greater challenges to manage than those selected at greater than MIC (Andersson and Hughes 2012). They increase complications in infection treatment (Andersson and Hughes 2010) and remain a public health concern (Capita et al. 2014). They do not lose their resistance traits in the absence of antimicrobials, are more stable, and promote enrichment of resistance (Andersson and Hughes 2010, 2012). At lower concentrations, the risk of emergence of resistant populations in the environment not only increase (Knapp et al. 2008; Couce and Blazquez 2009), but the problem of horizontal gene transfer to other populations could intensify (Couce and Blazquez 2009; Canton and Morosini 2011; Johnson et al. 2015), which includes induced transfer of plasmids and transposons (Barr et al. 1986; Doucet-Populaire et al. 1991), and enhanced recombination (Lopez et al. 2007; Lopez and Blazquez 2009). Increased rates of replication (Andersson and Hughes 2009, 2011) and mutation (Cortes et al. 2008; Morero et al. 2011; Thi et al. 2011; Gutierrez et al. 2013; Chow et al. 2015) have also been evidenced. Moreover, low concentrations contribute to signalling molecules for biofilm formation and gene expression (Andersson and Hughes 2014; Aka and Haji 2015; Ebrahimi et al. 2015). As such, sub-lethal concentrations could stimulate the spread of resistance in the environment and increase the likelihood of multi-resistant bacteria through genetic changes (Sandegren 2014).

There are multiple factors that can influence the MSC of disinfectants. In the presence of a complex microbial community, selective forces that can change the selectability of any population at sub-MIC levels include nutrient concentrations, pH, and predation (Quinlan et al. 2011; Bengtsson-Palme et al. 2014b). A MSC model works best for planktonic bacteria growing in suspension form, rather than biofilm bacteria, as the presence of extracellular matrix interferes with chemical concentrations in the biofilm (Canton and Morosini 2011). Furthermore, selection of resistance does not depend on the initial number of resistant organisms in the system, and any resistant organism could become enriched in a community (Gullberg et al. 2011).

While sub-lethal concentrations of antibiotic have been studied (e.g., Bengtsson-Palme and Larsson 2016), the impact of disinfectants and their residuals has not been extensively investigated (Li et al. 2016). The purpose of this study is to examine the selection of resistant bacteria (Khan et al. 2016a) versus susceptible bacteria at specific sub-inhibitory concentrations of chlorine, either as free chlorine or monochloramine. Growth rates of susceptible and resistant bacteria were compared at different concentrations of disinfectants below the MICs of susceptible and resistant bacteria. We examined the merit of using minimum selectability concentration (MSC) as a toxicological approach to assess the emergence of antimicrobial resistant bacteria in the environment.

## Materials and methods

### Bacterial strains

Eight bacteria, belonging to four genera: *Bacillus*, *Paenibacillus*, *Acidovorax* and *Micrococcus*—previously isolated from drinking water environments (Khan et al. 2016b)—were chosen for this study. These bacteria were previously classified into three groups: resistant (R), intermediate (I), and susceptible (S) on the basis of size of zone of inhibition against disinfection with 14.5% standard sodium hypochlorite by disk diffusion method (Khan et al. 2016a). Closely related bacteria were paired together and used irrespective of their antibiotic resistance (Table 1).

**Table 1:** Mean minimum inhibitory concentrations of test micro organisms against free chlorine and monochloramine (*n* = 3)

Organisms	MIC (mg L^−1^ ± SD)^b^		Zone of inhibition (mm) against 14.5% standard NaOCl^a^
	Free chlorine	Monochloramine	
*Bacillus* sp. (R1)	10.4 ± 1.7	10.0 ± 3.8	8
*Bacillus* sp. (R2)	1.0 ± 0.6	5.0 ± 1.7	19^b^
*Paenibacillus* sp. (R)	10.0 ± 1.4	5.2 ± 1.6	20
*Paenibacillus* sp. (S)	5.2 ± 2.9	2.2 ± 1.1	54
*Acidovorax* sp. (R)	8.2 ± 2.0	8.2 ± 2.0	8
*Acidovorax* sp. (S)	2.0 ± 1.2	5.2 ± 1.6	50
*Micrococcus* sp. (I)	8.0 ± 3.1	4.8 ± 2.2	35
*Micrococcus* sp. (S)	5.0 ± 1.7	2.1 ± 1.2	48

*R* resistant, *I* intermediate, *S* susceptible

^a^ Unless otherwise stated, values were from Khan et al. (2016a)

^b^ Determined in this study

Bacteria were cryo-preserved (Cryo vials TS/71-MX, Technical Service Consultants Ltd. UK) and stored at −80 °C. For each experiment, a single bead of inoculum was aseptically removed from the cryovials, grown in LB broth (Oxoid, UK) overnight, and streaked on Nutrient Agar (Oxoid, UK) plates to generate isolates, which were used in the experiments. All bacteria were identified by 16S-rRNA gene sequencing (Khan et al. 2016a), except *Bacillus subtilis* (R2), which was acquired from culture collection (National Collection of Type Cultures, UK; NCTC 10400).

### Viable cell count by turbidity (OD_600_) measurement (Standard growth curve)

Cell concentrations were determined by spectrophotometrically, measuring the turbidity of solutions at 600 nm. To determine the relationship between OD_600_ (spectrophotometric optical density) and bacterial cell count (another microbiological measure of population), each bacterial isolate was grown overnight for maximum cell viability in 50-mL LB broth at 200 rpm on a shaker (Bench top Standard Analog, Orbital Shaker, VWR, UK) at 20 °C. Next day, the culture was concentrated by centrifuging (refrigerated centrifuge, Eppendorf, UK) three times at 3500 rpm for 10 min, and suspended in 0.1% PBS in a total volume of 5 mL. This culture was used to make ten-fold serial dilutions from 1:10 to 1:10,000, and two-fold serial dilutions from 1:2 to 1:128. OD_600_ of each dilution was recorded with a UV-VIs spectrophotometer (Helios Zeta, Thermo Scientific, UK) by taking 4 mL from each dilution tube in a 1 cm wide cuvette. Sterile PBS (0.1%) was used as blank. For the determination of number of bacteria (cfu mL^−1^) at a specific OD, the dilution tubes were further diluted up to 1:10,000 in 10 mL PBS whenever required, and 100 µL from the last dilution tube was transferred to Mueller Hinton Agar plates (Oxoid, UK) in duplicate, spread with a sterile spreader and incubated for 24 h at 35 ± 2 °C for the development of colonies. After 24 h, colonies were counted on each plate and cfu mL^−1^ was calculated for each OD_600_ and dilution. *Ln*(OD_600_) vs. *ln*(cfu mL^−1^) graph values were used for plotting and for the calculation of number of bacteria present at a specific OD in further experiments (Hall et al. 2014).

### MIC determinations for free chlorine

Experiments were performed in 50-mL screw- capped glass vials in a total volume of 10-mL PBS, pH 7.0. Glass vials were pre-treated with 10% HNO_3_ (prepared from 69%, AnalaR NORMAPUR, Prolabo VWR BDH) overnight, soaked in 1% NaOCl (Alfa Aesar, UK), rinsed with nano-pure water (18Ώ), and sterilized before use. Bacterial strains were grown overnight in LB broth with continuous shaking at 200 rpm at 20 °C, and washed three times with PBS (﻿﻿pH 7.0) to remove organic material. Bacterial stock culture was suspended in the same buffer, and diluted to a turbidity between 0.08–0.13 at OD_600_, equivalent to a bacterial concentration of 1–1.5 × 10^8^ cfu mL^−1^. Chlorine solutions were prepared freshly at the time of each experiment, having concentrations of 0.001 L^−1^ to 10 mg L^−1^ from a standard stock solution of 14.5% sodium hypochlorite (Alfa Aesar, UK) in chlorine-demand free PBS. Bacterial stock culture was diluted, added at a concentration of 1 × 10^5^ cfu mL^−1^ in the vials, and vials were incubated for 24 h at 37 °C. After incubation, 1 mL of the solution from each vial was spread with a sterile spreader on to Mueller Hinton Agar plates (Oxoid, UK) in duplicate, and plates were incubated for 24–48 h at 37 °C for the development of colonies. The lowest concentration of free chlorine without any sign of growth on representative plates after 48 h was considered as the MIC of free chlorine against that organism (Clinical and Laboratory Standards Institute 2012). With this measure, the concentrations with the appearance of colonies were considered non-inhibitory for the organism. The experiments were run in triplicate on three different days to determine the minimum inhibitory concentration (MIC) of disinfectant.

### MIC determinations for monochloramine

For monochloramine experiments, PBS of pH 8.0 was used. Monochloramine solutions were prepared by mixing the appropriate volume of 1.91% NH_4_Cl (Sigma-Aldrich, UK) and 14.5% NaOCl (Alfa Aesar, UK) solutions. A series of monochloramine concentrations from 0.001 to 10 mg L^−1^ were prepared in PBS. The remaining protocol was the same as that used for chlorine (described above).

### Selection of medium for growth rate experiment

For the determination of µ_max_ (ultimate population growth rate) and appropriate growing media, experiments were carried out in different concentrations of LB broth, 0.1, 1.0, 5.0, 10, and 100%, and 10 mM PBS (pH: 7.0; representing 0% LB) in sealed serum vials. Hundred millilitre broths and PBS were inoculated with overnight grown cultures of *Bacillus* (R1 and R2) and *Paenibacillus* (R and S) species at a concentration of 1 × 10^6^ cfu mL^−1^, and allowed to grow with continuous shaking at 20 °C. Optical densities (OD_600_) were measured over 96 h (6 h intervals) with a UV-VIs spectrophotometer (Helios Zeta, Thermo Scientific, UK). Growth rate was calculated from the plots of *ln*(OD_600_) vs. time. Media was selected on the basis of bacterial growth and low chlorine demand, while bacteria were selected on the basis of oxygen requirement; two genera were used. *Bacillus*, *Micrococcus* and *Acidovorax* are aerobic, so *Bacillus* was selected as representative, while *Paenibacillus* was the only facultative anaerobe, so it was included in this experiment. PBS had minimum chlorine demand but tested bacteria showed negative growth rate so they were not used for further experiment. LB broth (0.1%) was selected as a medium for growth for further experiments of MSC of disinfectants as it had low chlorine demand and bacteria grow well in the broth.

### Preparation of bacterial inoculum for growth rate experiments

Cryo-preserved culture, previously stored at −80 °C, was grown in LB broth overnight, and streaked on Mueller Hinton Agar plates (Oxoid, UK) to verify culture purity. A single colony was transferred to 20 mL LB broth in a sealed glass bottle and grown overnight at 20 °C to obtain log phase culture with a high viable count. The oxygen environment in the glass bottle was representative of conditions in water distribution pipes and allowed relatively rapid growth in fresh medium without excessive chlorine demand. This culture was washed three times with chlorine demand free 0.1% LB, and suspended in the same broth for growth rate experiments (Berney et al. 2006; Hall et al. 2014). Chlorine demand of the broth was calculated by the formula; chlorine demand = chlorine added concentration (mg L^−1^)—chlorine residual concentration (mg L^−1^) after 30 min contact time (HACH methods 10069 and 10223, DPD reagent, HACH, UK).

### Growth rate experiments with disinfectants for MSC

Experiments were performed in 0.1% LB broth in 100 mL sterile sealed serum vials to avoid the evaporation of chlorine. Free chlorine solutions of 10 different concentrations 0.01, 0.02, 0.03, 0.04, 0.05, 0.1, 0.5, 1.0, 5.0 and 10 mg L^−1^ were prepared as target concentrations in dilute LB. Overnight grown culture (as describe above) was diluted and added at a concentration of 1 × 10^8^ cfu mL^−1^ in the final volume of 100 mL, and vials were sealed immediately and mixed well. The vials were incubated at 20 °C with continuous shaking at 200 rpm for 24 h, and OD_600_ were taken with a UV-VIs spectrophotometer at 2 h time intervals by removing 4 mL medium from each vial.

The growth rate constant (µ) was calculated for each bacterium from the previously determined growth curve (OD_600_ vs. cfu mL^−1^, previous section) by converting the OD_600_ into cfu mL^−1^ and calculating the µ by the slope of the graph between *ln*(cfu mL^−1^) vs. time. The experiments were run in triplicate for each concentration and mean growth rate constant was determined.

### Data analysis

Concentrations were log transformed before analysis. Statistical analysis was carried out using Minitab-v17. Correlations were determined between concentrations of the two disinfectants and growth rates by Pearson’s Correlation test (*p* = 0.05) (Table 2). Minimum selectable concentrations (MSCs) were determined from growth rates vs. concentrations (log_10_ transformed) plots where the growth rate of resistant bacteria exceeded that of the susceptible population. Non-linear regression was performed using GraphPad Prism version 7.01 for Windows (GraphPad Software, La Jolla, CA, USA) to calculate the MSC values from standard curves at 95% confidence interval.

**Table 2:** Correlation between growth rates and concentrations (*log* transformed) of free chlorine and monochloramine by Pearson correlation test (*α* = 0.05)

Disinfectant	Organism	*R*-value	*P*-value
Chlorine	*Bacillus sp. R1*	−0.959	<0.001
	*Bacillus sp. R2*	−0.893	0.001
	*Paenibacillus sp.*	−0.954	<0.001
	*SPaenibacillus sp. R*	−0.977	<0.001
	*Acidovorax sp. R*	−0.843	0.002
	*Acidovorax sp. S*	−0.760	0.011
	*Micrococcus sp. I*	−0.958	<0.001
	*Micrococcus sp. S*	−0.976	<0.001
Monochloramine	*Bacillus sp. R1*	−0.905	<0.001
	*Bacillus sp. R2*	−0.941	<0.001
	*Paenibacillus sp.*	−0.961	<0.001
	*SPaenibacillus sp. R*	−0.962	<0.001
	*Acidovorax sp. R*	−0.912	<0.001
	*Acidovorax sp. S*	−0.943	<0.001
	*Micrococcus sp. I*	−0.978	<0.001
	*Micrococcus sp. S*	−0.926	<0.001

## Results

### Zone of inhibition by selected bacterial strains

Eight bacteria (four different genera) were selected for this study. They were divided into three groups on the basis of size of zone of inhibition (in diameter); Resistant (R) ≤ 20 mm, Intermediate (I) = 21–40 mm, and Susceptible (S) ≥ 41 mm, as described previously (Khan et al. 2016a). One member of each pair had a zone <20 mm, while the second member had a zone ≥41 mm, except for *Bacillus and Micrococcus* spp.; both *Bacillus* produced <20 mm zones and were differentiated by R1 and R2, while *Micrococcus* spp. produced 35 and 48 mm zones of inhibition and were differentiated by I and S, respectively. Other bacteria included *Paenibacillu*s spp. having 20 and 54 mm zones, and *Acidovorax* having 8 and 50 mm zones, respectively (Table 1).

### MIC of the bacterial strains against chlorine and monochloramine

Bacteria were tested by dilution method against a series of concentrations of free chlorine and monochloramine from 0.01 to 100 mg L^−1^ to determine the MICs of these disinfectants against the eight microorganisms. The MICs of free chlorine and monochloramine were in the ranges from 1–10.4 mg L^−1^, and 2.1–10 mg L^−1^, respectively (Table 1). *Bacillus* sp. (R1) showed the highest MICs for free chlorine and monochloramine, which were 10.4 ± 1.7 and 10.0 ± 3.8 mg L^−1^, respectively. *Bacillus* sp. (R2) showed lowest MIC 1.0 ± 0.6 mg L^−1^ for chlorine, while *Micrococcus* sp. (S) had lowest MIC 2.1 ± 1.2 mg L^−1^ for monochloramine (Table 1).

### Selection of suitable medium for growth rate experiment

Growth rates of *Bacillus* and *Paenibacillus* spp. were tested at six different concentrations of LB broth, and were observed in the range of −0.076 to 0.462 h^−1^ in these media (Table 3). PBS (10 mM) showed minimum growth rate and chlorine demand, but *Paenibacillus* sp. (S) did not grow well in PBS, so 0.1% LB broth was selected for the further experiments; it had lowest chlorine demand, whilst supporting bacterial growth.

**Table 3:** Growth rates of selected bacteria in different growth medium

Organisms	Growth rates in growth medium (h^−1^)					
	10 mM PBS	0.1% LB	1% LB	5% LB	10%LB	100% LB
*Bacillus* sp. (R1)	0.035 ± 0.01^b^	0.261^c^	0.224^c^	0.424^c^	0.181 ± 0.20^a^	0.304 ± 0.14^a^
*Bacillus* sp. (R2)	0.099 ± 0.10^b^	NT	NT	NT	0.462 ± 0.34^b^	0.237 ± 0.03^b^
*Paenibacillus* sp. (R)	0.028 ± 0.00^b^	0.197^c^	0.218^c^	0.283^c^	0.326 ± 0.16^a^	0.343 ± 0.28^a^
*Paenibacillus* sp. (S)	−0.076 ± 0.18^b^	NT	NT	NT	0.015 ± 0.21^b^	0.127 ± 0.13^b^

*NT* not tested

^a^ n=3

^b^ n=2

^c^ n=1

### Minimum selectable concentration (MSC) of disinfectants

**Fig. 2:**
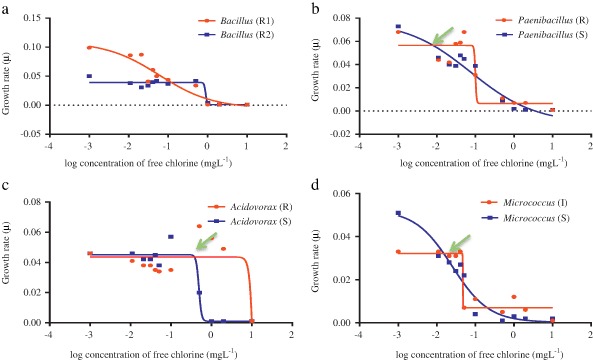
Minimum selectability concentrations (MSC) of *Bacillus* (**a**), *Paenibacillus* (**b**), *Acidovorax* (**c**), and *Micrococcus* (**c**) species for free chlorine

**Fig. 3:**
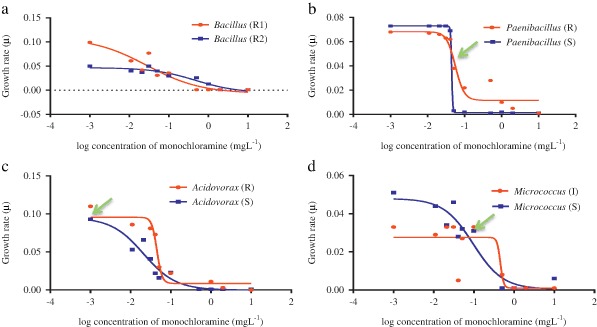
Minimum selectability concentrations (MSC) of *Bacillus* (**a**), *Paenibacillus* (**b**), *Acidovorax* (**c**), and *Micrococcus* (**d**) species for monochloramine

Bacteria, in their log phase of growth, were exposed to a series of concentrations (0.01–10 mg L^−1^) of free chlorine and monochloramine, and their growth rate constants (µ) were compared (Figs 2 and 3). Minimal selectable concentration (MSC) represented the sub-MIC concentration at which the more resistant organism’s growth exceeded its competitor. Each bacterial pairing showed different behaviour with the different disinfectants—free chlorine and monochloramine. The *Micrococcus* assay showed the greatest difference between MIC (5.0 ± 1.7 mg L^−1^) and MSC (0.046 mg L^−1^), which was 110 fold lower than the MIC of the susceptible strain against chlorine (Table 4). While with monochloramine, *Acidovorax* assay MIC/MSC was more than any other bacteria. MSC was 0.021 mg L^−1^ which was 1/250th the MIC value of the susceptible organism. The non-linear regressions fitted data point well, with consistent *R* ^2^ > 0.90 and *S* (Standard Error of Regression) < 0.010; exceptions were *Acidovorax* (S) in the chlorination experiment (*R* ^2^ = 0.64; *S* = 0.011), and *Micrococcus* (I) (*R* ^2^ = 0.72; *S* = 0.009) in the chloramine experiment. Details of MSC and its ratio with MIC can be found in Table 4. The MSC for *Bacillus* against chlorine and monochloramine could not be calculated from the data, since the results suggested that the resistant strain had competitive advantage at much lower concentrations used in this study making determination difficult.

**Table 4:** Minimum selectable concentrations (MSC) of free chlorine and monochloramine for bacteria isolated from water distribution systems

Organism	Free chlorine				Monochloramine	
	MSC mg L^−1^	MIC_*susc*_ mg L^−1^	MIC_*susc*_/MSC	MSC mg L^−1^	MIC_*susc*_ mg L^−1^	MIC_*susc*_/MSC
*Bacillus*	NC	1.0	NC	NC	5.0	NC
*Paenibacillus*	0.089	5.2	58.4	0.046	2.2	47.8
*Acidovorax*	0.393	2.0	5.1	0.021	5.2	247.6
*Micrococcus*	0.046	5.0	108.7	0.319	2.1	6.6

*NC* not calculated

## Discussion

Sub-lethal concentrations of antimicrobials can create conditions that selectively favour more resistant organisms (Chow et al. 2015). The enrichment of resistant bacteria can occur at concentrations many fold below the MIC_*susc*_(Hughes and Andersson 2012). In this study, the bacteria pairings had similar growth rates at very low chlorine and chloramine concentrations. Once chemical concentrations exceeded a particular threshold (the MSC), the growth rate of the more susceptible population declined as compared to resistant population.

The relevance of the study suggests that we should also be concerned about the MSC than just the MIC when examining antimicrobial resistance. Natural environments, which can be exposed to relatively low concentrations of antimicrobials, are also prone for the enrichment of resistance (Drlica 2003; Drlica and Zhao 2007), as well as high-concentration exposures (Myers 2008). This is also relevant along concentration gradients from a point of high-exposure (e.g., over time for a degrading compound, or spatially when dispersed). For example, in drinking water treatment plants, high concentration i.e. 0.5 mg L^−1^ or more of residual disinfectant is applied to the system, but by the time the water reaches the point of use, the concentration may have reduced to sub-inhibitory levels i.e. less than 0.1 mg L^−1^, as found in this study because of the short half-life of these disinfectants. This concentration gradient could increase the selection of resistant populations (Zhou et al. 2000) if bacterial contamination is allowed to enter the system. This could also become relevant to downstream areas where chlorinated water supplies discharge into the natural environment. Thus, the presence of sub-lethal concentrations of disinfectants increases the risk of dispersion of resistant bacteria through water distribution systems.

In this study, a series of concentrations of chlorine and monochloramine were used and enrichment of disinfectant resistant populations was observed in several cases (Figs 2 and 3), supporting the idea that low concentrations of chlorine and monochloramine could selectively enrich resistant bacteria. Similar results were obtained in a previous study where the selection of multidrug resistant *Ps. aeruginosa* was observed after treatment with sub-optimal concentration of chlorine (Shrivastava et al. 2004).

Several mechanisms could be responsible for resistance development against chlorine-based disinfectants at sub-MIC levels (Moen et al. 2012): increased surface hydrophobicity (Hostacka et al. 2003), changes in exopolymeric matrix (Dynes et al. 2009), detoxifying efflux genes (Mc Cay et al. 2010; Moen et al. 2012), differential expression of outer-membrane porin genes (Moen et al. 2012), morphological modifications, high enzyme activities (Gao and Liu 2014), transfer of conjugative plasmid carrying resistance traits (Johnson et al. 2015), and regeneration pathways (Drazic et al. 2015; Jozefczuk et al. 2010). A recent study showed that not only disinfectants, but their by-products, could also enrich resistant bacteria at sub-lethal concentrations through chromosomal genetic mutation in water (Lv et al. 2014; Li et al. 2016). Environmental conditions could also have multiplicative effects in the enrichment process; sub-inhibitory concentrations of benzalkonium chloride selects adaptive variants of *Ps. aeruginosa* in magnesium limited medium, but not in organic-carbon rich conditions (Mc Cay et al. 2010).

Different methods could be used for determination of minimal selective concentrations, such as use of mutant and wild type bacteria with different resistant markers and use of different fluorescence proteins to distinguish between sensitive and resistant populations (Gullberg et al. 2011). The growth rate approach offers an advantage over other techniques by not requiring additional markers to verify resistant populations. Comparing bacterial growth rates is considered an important tool for understanding microbial physiology (Hall et al. 2014). Bacterial growth rate data can be used in environmental studies for quantifying phenotypes (Warringer and Blomberg 2003), and their adaptation to environmental changes (Lindsey et al. 2013). In this study, growth-rate data were applied to determine minimal selective concentrations of disinfectants which lead to increased resistance traits. It has been considered that the disinfectant resistance could enhance antibiotic resistance in environment and contribute to increased public health risk (Al-Jailawi et al. 2013; Capita et al. 2014; Seier-Petersen et al. 2014).

## Conclusion

Seven drinking-water isolates and a single culture-collection strain were exposed to varying levels of chlorinated disinfectants. Results found that lower than expected concentrations (i.e., <MIC, a conventional metric for bacterial resistance) showed selective bias by providing resistance strains a competitive advantage in population growth. It is important to recognise sub-lethal effects of disinfectants on resistant strains because of their potential impact on drinking water contamination and human health. In the environment, sub-MIC levels of disinfectants are present as residuals which could select resistant bacteria and potentially facilitate the dissemination of resistant determinants among bacteria. There is a need for further investigation to understand the ecological responses of bacteria in the presence of sub-MIC level of disinfectants (and antibiotics) to overcome the problem of enriched antimicrobial-resistant (antibiotic resistant) populations that have become a concern on a global scale. Broadening ecotoxicological studies to strategically include selectivity metrics, e.g., MSC, would be an important step forward.
